# Hypertension and Diabetes: Entry Points for Prevention and Control of the Global Cardiovascular Epidemic

**DOI:** 10.1155/2013/878460

**Published:** 2013-04-07

**Authors:** Shanthi Mendis, Eoin O'Brien, Yackoob Kassim Seedat, Salim Yusuf

**Affiliations:** ^1^Management of Noncommunicable Diseases, World Health Organization, Geneva 1211, Switzerland; ^2^Conway Institute of Biomolecular and Biomedical Research, University College Dublin, Ireland; ^3^Nelson R Mandela School of Medicine, University of KwaZulu-Natal, Congella, Durban, South Africa; ^4^Population Health Research Institute, McMaster University, Canada

 Complications of hypertension are associated with an estimated 9.4 million deaths worldwide every year [1]. In 2008, globally, the overall prevalence of hypertension (including those on medication for high blood pressure) in adults aged 25 and over was around 40% [2]. On average, global population systolic blood pressure decreased slightly between 1980 and 2008 [3, 4], although the worldwide prevalence of obesity has nearly doubled during this period. In 2008, the global prevalence of high cholesterol was 40% and prevalence of diabetes was 10% in adults over 25 years [1]. 

Most people with diabetes and hypertension also have other cardiovascular risk factors such as raised lipids [1, 2]. To reduce the prevalence and consequences of hypertension and diabetes a complimentary mixture of population-wide and individual interventions is required. To ensure optimal coverage of the population with these interventions implementation of public health policies has to be complimented with a health system which addresses hypertension through affordable strategies [2, 5]. An approach that relies mainly on the overall risk of individuals is likely to be more cost effective than one focused solely on blood pressure levels or targets.

There are many barriers to the control of hypertension and diabetes in low- and middle-income countries. They include the double burden of communicable and noncommunicable diseases, inadequate investment in health and prevention, fragile health systems particularly at primary care level, and lack of or maldistribution of health workers. Several countries spend less than 50 USD per capita per year on health. This low level of investment is inadequate to effectively address noncommunicable diseases in a sustainable manner [2]. 

To address cardiovascular disease, diabetes, and noncommunicable diseases a set of core interventions (Table 1) have been identified which are highly cost effective, affordable, and feasible to implement even in resource-constrained settings [6]. These interventions address diabetes, hypertension, and their key underlying risk factors—unhealthy diet, harmful use of alcohol, and physical inactivity. Some of these interventions are feasible in primary care even in low-resource settings. For example, people at risk of heart attacks and stroke usually have a modest elevation of multiple risk factors, such as smoking, raised blood pressure, raised cholesterol, and/or diabetes. Such people who have medium or high cardiovascular risk can be treated with a multidrug regimen and behavioral modification to reduce the risk of developing future heart attacks, strokes, cardiac failure, and kidney disease. This integrated intervention applied to individuals with an overall moderate or high cardiovascular risk based on integrating risk based on age or several risk factors is more cost effective than conventional vertical approaches to single-risk-factor interventions [5]. 

Further, the development and deployment of a cadre of nonphysician health workers, by removing cultural and legal barriers, will facilitate screening for hypertension and diabetes. This can lead to prescribing a limited number of safe, proven, and affordable medicines by nonphysician health workers [7, 8]. Protocols and tools to estimate cost of implementation have also been developed to facilitate delivery of these very cost-effective interventions [9, 10]. WHO estimates show that to implement the interventions listed in the table for all low- and middle-income countries, the cost of implementation per head of population is low [6]. It amounts to an annual investment of under US$ 1 in low-income countries, US$ 1.50 in lower middle-income countries, and US$ 3 in upper middle-income countries [6]. Expressed as a proportion of current health spending, the cost of implementation amounts to 4% in low-income countries, 2% in lower middle-income countries, and less than 1% in upper middle-income countries [4]. 

The special issue aims to highlight solutions that are designed to address the global cardiovascular epidemic. 

It contains three original research articles and one review. S. Tiptaradol and W. Aekplakhorn estimated the prevalence of coexistence of diabetes and hypertension and the proportion of awareness, treatment, and control of both conditions using data from Thai National Health Examination Survey III. They found that about half of the diabetes patients also had hypertension, conversely about 14% of hypertensive patients had diabetes, and that more than 80% were unaware of having both conditions. 

M. Chiha and colleagues focused on type 2 diabetes mellitus as a risk factor for coronary heart disease. They summarize the mechanisms of atherogenesis in diabetes, the impact of hypertension, the treatment goals in diabetes, and the epidemiologic consequences of diabetes and heart disease on a global scale. They reported that diabetes mellitus is associated with an increased risk of cardiovascular death and a higher incidence of cardiovascular diseases including coronary artery disease. They highlighted the need for appropriate screening to help better manage the cardiovascular events in people with diabetes. I. Codreanu and others showcased the International Society of Nephrology (ISN), Global Outreach Program, aimed at building capacity for detecting and managing chronic kidney disease and its complications in low- and middle-income countries using the work done in the Republic of Moldova. They concluded that individuals with hypertension and diabetes should be screened for the coexistence of renal abnormalities, with the intention of developing disease-specific healthcare interventions to reduce CV morbidity and mortality and prevent renal disease progression. S. Mendis and colleagues reported on a cross-sectional study conducted in eight low- and middle-income countries to evaluate the capacity of primary care facilities to implement basic interventions to address major noncommunicable diseases, including cardiovascular diseases and diabetes. They identified major deficits in health financing, access to basic technologies and medicines, medical information systems, and the health workforce. 

This special issue is released at an opportune moment because the theme of the World Health Day on April 7, 2013, is focusing on hypertension to prevent heart attacks and strokes. The issue reiterates the contribution of raised blood pressure and diabetes to the global cardiovascular disease burden, the necessity to recognize the close association between hypertension, diabetes, renal disease, and other risk factors on health outcomes, and the need to strengthen health systems in low- and middle-income countries in order to scale up prevention and control of hypertension, diabetes, and other noncommunicable diseases. 

As countries develop the Action Plan for Prevention and Control of Noncommunicable Diseases 2013–2025 in response to the recommendations of the Political Declaration of the United Nations General Assembly on NCDs [11], an emphasis on affordable interventions is essential to efficiently tackle the growing burden from NCDs, particularly in low- and middle-income countries. 

*Shanthi Mendis* *Shanthi Mendis* *Eoin O'Brien* *Eoin O'Brien* *Yackoob Kassim Seedat* *Yackoob Kassim Seedat* *Salim Yusuf* *Salim Yusuf*

## Figures and Tables

**Table 1:** A core set of very cost-effective interventions for prevention and control of noncommunicable diseases including cardiovascular disease [6].

NCD core intervention set (best buys)		
	Reducing tobacco use	(i) Excise tax increases; (ii) smoke-free indoor workplaces and public places; (iii) health information and warnings about tobaco; (iv) bans on advertising and promotion
Population-based interventions addressing NCD risk factors	Reducing harmful alcohol use	(i) Excise tax increases on alcoholic beverages; (ii) comprehensive restrictions and bans on alcohol marketing; (iii) restrictions on the availability of retailed alcohol
	Promoting healthy diets and promoting physical activity	(i) Salt reduction when high, reduced salt content in processed foods; (ii) replacement of transfats with polyunsaturated fats; (iii) public awareness media campaign about diet and physical activity

Individual-based interventions addressing NCDs in primary care	Reducing complications in individuals with CVD and diabetes	(i) Drug therapy (including glycaemic control for diabetes mellitus) to individuals who have had a heart attack or stroke, and to persons with a high risk (>30%) of a CVD event in the next 10 years; (ii) providing aspirin to people having an acute heart attack.
